# Evaluation of Copper-Based Alloy (C93200) Composites Reinforced with Marble Dust Developed by Stir Casting under Vacuum Environment

**DOI:** 10.3390/ma12101574

**Published:** 2019-05-14

**Authors:** Santosh Kumar Rajak, Amit Aherwar, Deepak Rajendra Unune, Mozammel Mia, Catalin I. Pruncu

**Affiliations:** 1 Department of Mechanical Engineering, Madhav Institute of Technology and Science, Gwalior-474005, India; skrajak.sdl@gmail.com (S.K.R.); amit.aherwar05@gmail.com (A.A.); 2 Department of Mechanical-Mechatronics Engineering, The LNM Institute of Information Technology, Jaipur-302031, India; deepunune@gmail.com; 3 Mechanical and Production Engineering, Ahsanullah University of Science and Technology, Dhaka 1208, Bangladesh; mozammelmiaipe@gmail.com; 4 Mechanical Engineering, Imperial College London, Exhibition Rd., London SW7 2AZ, UK; 5 Mechanical Engineering, School of Engineering, University of Birmingham, Birmingham B15 2TT, UK

**Keywords:** C93200 alloy, marble dust, PSI method, multi-criteria decision-making

## Abstract

Copper-based alloy (C93200) composites reinforced with a different weight percentage of marble dust particles (1.5, 3, 4.5, and 6 wt.%) were developed by stir casting method under vacuum environment. By using this type of reinforcement, it was possible to detect a suitable material for bearing applications. The manufactured material was characterized for its mechanical properties using a micro-hardness tester. A universal INSTRON-5967 machine was used to detect the yield and tensile strength. Further the hardness features were measured using a Walter Uhl model machine, whereby the wear characteristics were simulated under the pin-on-disc tribometer under different working conditions in ambient temperature (23 °C). Next, the preference selection index (PSI) technique that considers multi-criteria decision-making was proposed to validate which material was the best candidate. For the selection of material criteria, some specific material intrinsic properties—such as, density, void fraction, hardness resistance along with tensile, compressive, and flexural strength—were proposed and the surface characteristics linked to friction coefficients along wear properties. It was found that the novel composite material containing 4.5 wt.% of marble dust provided the best combination of properties and is a suitable candidate material for bearing applications.

## 1. Introduction

Today, the global market is very competitive. Therefore, detecting an appropriate candidate material that meets the target of industrial application is a major challenge for researchers and design engineers. An improperly chosen material may not be solely responsible for the premature failure of components, but it may negatively affect the manufacturing process, productivity, profit, and deplore the name of reputed manufacturing organizations in the marketplace [1]. Numerous advanced engineering materials have been developed during the past few decades. Due to superior physical, mechanical, and tribological properties, metal composite has also been considered as a class of advanced engineering material, and it is being used for high-performance applications such as those in aerospace and automobile industries [2,3].

Nowadays, particle reinforced metal composites are being extensively utilized for properties such as a higher specific strength and elastic modulus, a minimum coefficient of thermal expansion, higher wear resistance, and a better performance at elevated temperatures. Furthermore, ceramic particulates such as Al_2_O_3_, SiC, MgO, and whiskers/fibers are mostly used as a reinforcement to improve mechanical and tribological properties through the proper selection of several parameters, such as the volume/weight percentage, shape and size of the reinforcement particles, and the dispersion of reinforcement in the matrix phase [4]. Poddar et al. [5] studied AZ91D composites reinforced with silicon carbide (SiC) particulates and they observed that the presence of particulates increases the yield strength and Young’s modulus because tiny particle reinforcement results in the improvement of the elastic modulus and yield strength. The mechanism of composite deformation is the load transfer from the matrix to reinforcement and a good bonding between matrix and reinforcement provides better load transfer and an enhancement of the properties. The effect of the particle size on the physical and mechanical properties of SiCp/5210 Al metal matrix composite is studied by Dong et al. [1], who explain that the bending strength of the composites increases with the decreasing particle size. 

For each product, the design engineer must consider the fundamental properties of the material (i.e., physical, mechanical, tribological, chemical properties, respectively) in accordance with its light weight and raw material price in order to propose the best candidate material [4,5,6,7,8,9]. Each material has some unique properties and a unique performance. Therefore, more than one material from the industrial library meet all the desired properties for a specific application. The material selection process among the host of alternatives and various conflicting criteria can be considered an example of multi-criteria decision-making (MCDM) problems. 

In the recent past, plenty of work has been accomplished using MCDM techniques for selecting the best potential material option. These techniques are accepted tools to choose preeminent alternatives for given applications. The techniques are: AHP, GRA, SAW, TOPSIS, WPM, PROMETHEE, VIKOR, GTMA, etc. [7,8,9,10,11,12,13]. Shanian [14] applied the TOPSIS, multi-criteria decision-making method when searching for the optimal material. The results were compared with other MCDM methods to solve such challenging problems within highly sensitive parts. Rao [15] applied GTMA (graph theory and matrix representation approach) for the material selection. Thakker et al. [16] successfully combined three different techniques (i.e., value engineering techniques; the Cambridge Material Selector (CMS)-based approach, and TOPSIS) to sort out materials for impulse turbine blades. Rao [17] introduced a ‘material suitability index’ that permits evaluating and ranking the best option using the GTMA method. Chatterjee et al. [18] successfully implemented the VIKOR and ELECTRE-II technique that solves the problem of two material selection and also compares their relative performance. Athawale et al. [19] successfully designed the utility additive method to identify suitable materials. Shanian and Savadogo [20] implemented the ELECTRE (ELimination and Et Choice Translating Reality) approach. Manshadi et al. [21] projected a novel method that combines a non-linear normalization strategy with a modified digital logic strategy. Rao [22] used a compromise ranking strategy. Prasenjit et al. [23] implemented VIKOR (that simulate a compromise ranking) alongside ELECTRE (outranking methods). Other work proves that material selection can be obtained using fuzzy decision-making combined with a multi objective decision-making strategy [24,25,26].

Therefore, large numbers of research proposed innovative strategies for detecting the best candidate material by implementing different multi-attribute decision-making methods. Although no clear application for the preference selection index (PSI) technique has been found so far, this technique permits an efficient ranking solution in which quality is the main attribute. Hence, this method can be used where different conflicts occur in deciding the importance of different criteria. The overall preference attribute together with the preference selection index from each individual alternative allows for the best possible alternative with a higher rank order [27,28,29].

This study firstly presents details of the manufacturing process for a series of copper-based alloy composites reinforced with marble dust particles, and the developed material was experimentally characterized for its physical, mechanical, and tribo-performance. Next, the Preference Selection Index (PSI) strategy was implemented to identify the best candidate material from manufactured composites intended for bearing applications.

## 2. Experimental Details

### 2.1. Materials and Fabrication Details

**Figure 1:**
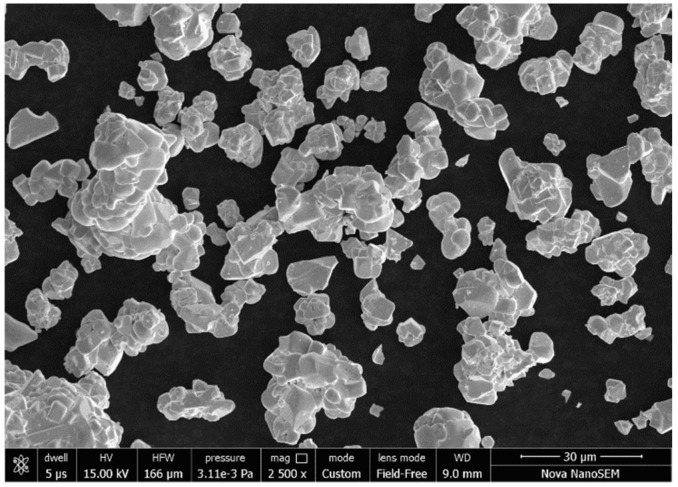
Scanning electron microscope (SEM) micrograph of marble dust powder.

C93200 copper alloy (ingot form) and marble dust in the size of <75 μm (see Figure 1) were procured from Mittal Industries, Jaipur, India. Different weight percentages (0, 1.5, 3, 4.5, and 6) of marble dust reinforced copper (C93200) alloy composites (A-1, A-2, A-3, A-4, and A-5, respectively) were developed by stir casting under a vacuum environment. The detailed composition of reinforcement (i.e., marble dust) included 40.84 wt.% LoI, 32.23 wt.% CaO, 18.94 wt.% MgO, 4.99 wt.% SiO_2_, and 3 wt.% other oxides, such as Al_2_O_3_, Fe_2_O_3_, SO_3_, K_2_O, and N_2_O. In Table 1 we present the designation and chemical composition of the studied C93200 copper alloy considered as the material matrix. The marble dust added C93200 copper alloy composites for bearing materials were fabricated by using liquid metal stir casting techniques in an induction furnace in a vacuum environment. The furnace consisted of a heating unit, graphite crucible, and a stirrer. Both the base alloy (i.e., C93200 copper alloy) together with the reinforcement (i.e., marble dust) was submitted to preheating (up to 400 °C) separately before starting the fabrication process. A graphite crucible was placed into the induction furnace to heat the base alloy. When the liquid temperature (around 1100 °C) was attained, a specific quantity of preheated marble dust was slowly added into the molten metal by a mechanical, continuous stirrer. The stirrer endured a constant rotational speed of 300 rpm for 2 min. that allowed the homogeneous mixing of filler material with the base (matrix) alloy. The wettability performances were ensured by incorporating, through the stirring process, a specific amount of magnesium (2 wt.%). Next, the mixture of base alloy and marble particles were poured into the graphite mould, which measured 100 × 65 × 10 mm^3^, followed by 15 min. cooling in air. Thus, five different weight percentages of marble particulate filled alloy composites were prepared. Thereafter, the casted specimens were polished and used for physical, mechanical, and wear characterization.

### 2.2. Mechanical and Physical Characterization

The Archimedes method, which assumes that immersion in fluid can obtain the weight of a specimen, was used to detect the material density. The theoretical density of C93200 alloy composites were later obtained by applying the mixture formula [30] as given in Equation (1).

(1) $$\rho_{th}=\frac{1}{\frac{w_{m}}{\rho_{m}}+\frac{w_{m1}}{\rho_{m1}}}$$

where $\rho_{m}$ and $\rho_{m1}$ represent the density of base alloy and density of marble and $w_{m}$ and $w_{m1}$ represent the weight fraction of base alloy and marble respectively.

The void fraction (*v_f_*) of fabricated composites can be calculated as following Equation (2).

(2) $$v_{f}=\frac{\rho_{th}-\rho_{\exp}}{\rho_{th}}$$

Micro-hardness tests were conducted (ASTM E92) with the help of the Walter Uhl model. The value of the applied load per capita was 100 gf for the dwell time of a 5-s period, and this process was repeated on five samples for each composite at different locations (to minimize the possible segregation effect during casting). The mean hardness value was calculated to ignore the variation of results. 

The tensile test, compression test, and three-point bending test of unreinforced and marble dust reinforced composites were conducted with the help of the INSTRON-5967 Universal testing machine (Norwood, MA, USA). The test samples were prepared according to the ASTM E8 standard. The tensile strength of specimens was carried out at a cross-head speed of 2 mm/min. Each type of test was repeated on five samples for each composite condition and the average value was reported. The compression test of the specimen with the dimension of 25 × 10 × 10 mm^3^ (cross-head speed of 2 mm/min) as per ASTM E9-09 standards was obtained on the Universal Testing Machine (UTM). The three-point flexural test of the specified composites was carried out on UTM-Instron (Norwood, MA, USA) conforming to ASTM-E290. 

### 2.3. Wear Test

The wear tests were conducted (as per ASTM G99) using a pin-on-disc tribometer, model no. TR 20, from Ducom Instruments, India. The pin-on-disc tribometer consisted of a pin holder, EN-31 hardened steel disc, an AC motor, and dead weight as per requirement. The composite pins (test coupon) were engaged and pressed constantly against the rotating EN-31 hardened steel disc (counterface) of hardness 60–70 HRC. Prior and after the sliding wear routine, the weight of the pins was measured by an electronic weighing machine with an accuracy of ± 0.001 mg. The difference in the mass between the initial and final weight of the pins indicated the mass loss (∆*m*) in the sliding routine. The analytical expression of wear loss is denoted by Equation (3).

∆*m* = *W*1−*W*2 (3)

where *W*1 and *W*2 represent the weight of the pins before and after wear routine, respectively, while ∆*m* is the entire mass loss in this routine.

## 3. The PSI Technique

**Figure 2:**
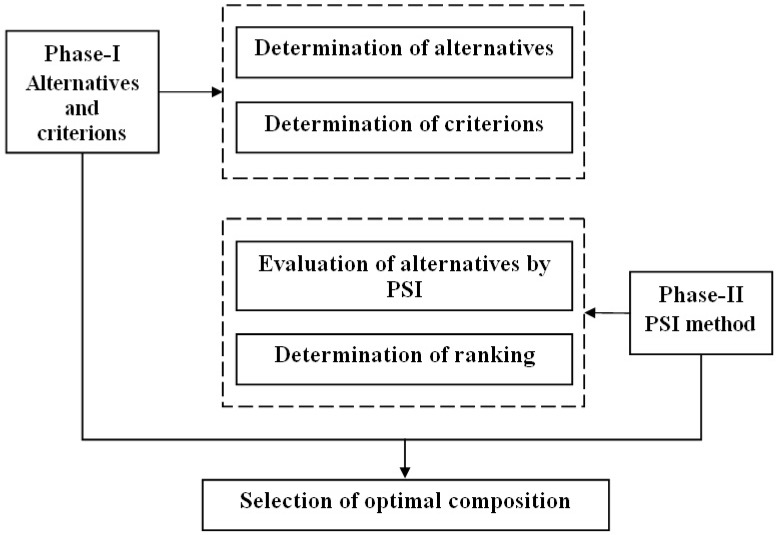
Optimization methodology for selection of the optimal composition.

The preference selection index (PSI) multi-criteriadecision-making technique was originally implemented to detect the best candidate material by Maniya and Bhatt [27]. Mostly, all the existing multi-criteria decision tools are based on the relative importance between its attributes or attribute weight that require countless calculations. By using the novel PSI method, it is possible to identify the best material without allocating the relative importance between its attributes. The beauty of this method is represented by the possibility of solving conflict generated by difficult decisions derived from the relative importance between its attributes. Once the preference value is established, a preference selection index linked to each alternative is calculated, while the highest value of PSI is generated as the best option. The entire schematic of the proposed MCDM model is given in Figure 2. 

The proposed MCDM model contains two main phases, namely:

Phase I Determination of alternatives and criterions
  In the initial state, we search for different numbers of alternatives and criteria for a specific MCDM problem.
Phase II Ordering the alternatives using Preference selection index (PSI) technique
  In this phase, the common steps are as follows:
    Step 1 Building the decision matrix: At this stage, in the PSI technique, we proceed to create a decision matrix that is derived from the alternatives and criteria imposed by the problem. If *m* represents the number of alternatives and *n* represents the numbers of criteria, a decision matrix of order *m* × *n* can be assembled and ascribed numerically by Equation (4).
      (4) $$T_{m\times n}=\begin{array}{cc} & \begin{array}{cccc} H_{1} & H_{2} & \cdots & H_{n} \end{array}\\ \begin{array}{c} B_{1}\\ B_{2}\\ \vdots \\ B_{m} \end{array} & \left[\begin{array}{cccc} T_{11} & T_{12} & \cdots & T_{1n}\\ T_{21} & T_{22} & \cdots & T_{2n}\\ \vdots & \vdots & \ddots & \vdots \\ T_{m1} & T_{m2} & \cdots & T_{mn} \end{array}\right] \end{array}$$
      here, each variable $T_{ij}$ (for *i* = 1, 2, 3, …, *m*; *j* = 1, 2, 3, …, *n*), from decision matrix, $T_{m\times n}$ represents the value of the potential alternative *i*th associated to a condition *j*th.
    Step 2 Decision matrix normalization: After constructing the decision matrix, their values were normalized in the range of [0,1] as described by Equation (5).
      (5) $$t_{ij}=\frac{T_{ij}}{T_{j}^{\max}},\;\mathrm{for}\;\mathrm{larger}\;\mathrm{is}\;\mathrm{good},\;\mathrm{and}\;t_{ij}=\frac{T_{j}^{\min}}{T_{ij}},\;\mathrm{for}\;\mathrm{smaller}\;\mathrm{is}\;\mathrm{good}$$
    Step 3 Determination of preference variation value: Once the decision matrix was normalized, we established the values of preference variation (Ψ*_j_*) applying a separate criterion that is solved by Equation (6).
      (6) $$\Psi_{j}=\sum_{i=1}^{m}{\left[t_{ij}-\frac{1}{m}\sum_{i=1}^{m}t_{ij}\right]}^{2}$$
    Step 4 Determination of deviation for each value from the preference variation: The values of deviation of the preference variation (Ω*_j_*) were determined applying Equation (7).
      (7) $$\Omega_{j}=1-\Psi_{j}$$
    Step 5 Calculations of the overall preference value: Once the deviation associated with the preference variation was determined is was possible to calculate the overall preference value (Φ*_j_*) by using Equation (8).
      (8) $$\Phi_{j}=\frac{\Omega_{j}}{\sum_{j=1}^{n}\Omega_{j}}$$
      The sum up of the overall preference values detected from the criterions can be described as i.e., $\sum_{j=1}^{n}\Phi_{j}=1$
    Step 6 Determination of the values for preference selection index: Once the overall preference values (Φ*_j_*) were computed, the preference selection index (Π*_i_*) associated to each alternative was calculated using Equation (9).
      (9) $$\Pi_{i}=\sum_{j=1}^{n}\left(t_{ij}\times \Phi_{j}\right)$$
      Finally, the alternatives were ranked in such way that the alternative with the highest Π*_i_* value was determined to be the best.

## 4. Results and Discussion

The values of density, void content, hardness, compressive mechanical strength, tensile strength, flexural strength, wear loss and COF under different conditions (normal loads of 10 N, 15 N, and 20 N, sliding velocities of 2.61 m/s, 3.66 m/s and 4.69 m/s), were gained and considered as criterions. Table 2 shows brief explanations of the measured criterions, and Table 3 represents the results obtained for five investigated composites.

### 4.1. Effect of Marble Dust Reinforcement on Criterion-1 and Criterion-2

**Figure 3:**
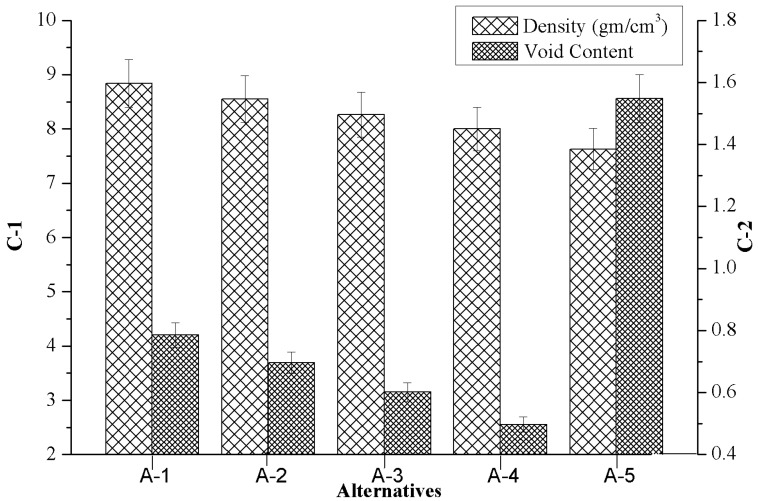
Variation of criterion-1 together with criterion-2 in respect to the values of alternatives.

Figure 3 presents the influence of the marble reinforcement on criterion-1 (density) and criterion-2 (void content) of composites. It was noted that the marble dust reinforcement produces a distinct transition for the composite’s characteristics. The present investigation aimed to develop the composite with a lower density (C-1) as well as void content (C-2) to achieve a high strength to weight ratio. Figure 3 shows that the density, as well as void content of composites, may have slightly decreased when the amount of alternatives (weight %) were increased. The reason for the subsidence in the void fraction appears to be due to an increase in wettability that promotes a nucleation process and crystal growth which results in less shrinkage porosity. On the other hand, the increase in the void fraction for the highest weight percentage of marble dust could be due to improper distributions of filler material i.e., segregation of reinforcement during the casting process. The lower density of composites was obtained for 6 wt.% of marble dust reinforcement (i.e., A-5) whereas the minimum void content was found for 4.5 wt.% of marble reinforcement (i.e., A-4). The subsidence in density and void content may be due to the lower density of marble dust and good bonding strength between the matrix alloy and reinforcement interface. The observations agree with the results presented in the literature by Gangwar et al. [31] when considering SiBr alloy reinforced with quicklime via high-temperature vacuum casting. 

### 4.2. Effect of Marble Dust Reinforcement on Criterion-3 and Criterion-4

**Figure 4:**
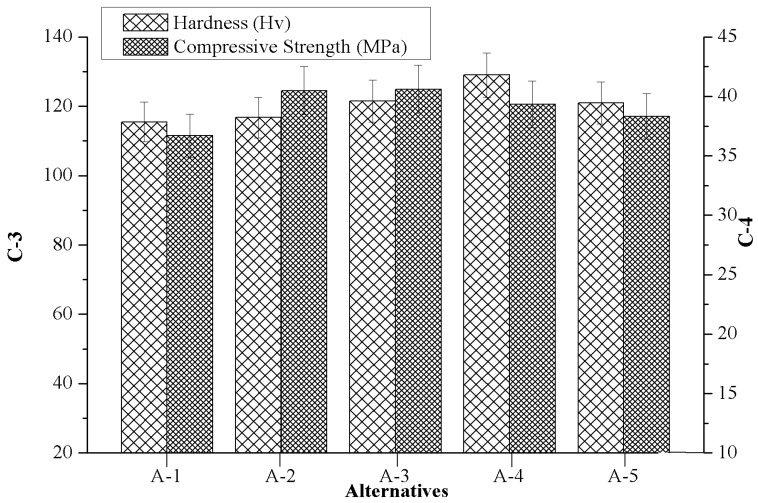
Variation of criterion-3 together with criterion-4 in respect to the values of alternatives.

Figure 4 depicts the effects of marble dust reinforcement in respect to criterion-3 (hardness) and criterion-4 (compressive strength) for the manufactured composites. As shown in Figure 4, when it is introduced in the composition of a composite with a higher marble content from 0 wt.% to 4.5 wt.%, the hardness of composites significantly improved from 115.49 Hv to a maximum of 128.97 Hv. Hereby, any further marble content introduced promoted a reduction in the hardness of composites up to 120.94 Hv (i.e., A-5). An improvement in the value of the microhardness of marble particulate C93200 alloy composites endorsed a better bonding strength achieved at the base alloy with a reinforcement interface. From Figure 4 we note that the compressive strength of the composite increased proportionally with the amount of marble content added and the maximum value of compressive strength was indentified within the composite that contained 3 wt.% of marble reinforcement (A-3). However, on further addition of marble particulate, the compressive strength of the composite gradually decreased. At a higher weight percentage of reinforcement, there is much possibility of segregation of reinforcement and less contact surface area between the matrix alloy and the reinforcement which resulted in a subsidence of hardness and compressive strength [31]. The other two reasons that may also be responsible for the decrement in the compressive strength of the composite, was caused by adding in different marble content: First, the effect of residual stress during the deformation process, and second, the failure of clustered submicron marble particles and presence of porosity and oxide layers during the casting process [32]. 

### 4.3. Effect of Marble Dust Reinforcement on Criterion-5 and Criterion-6

**Figure 5:**
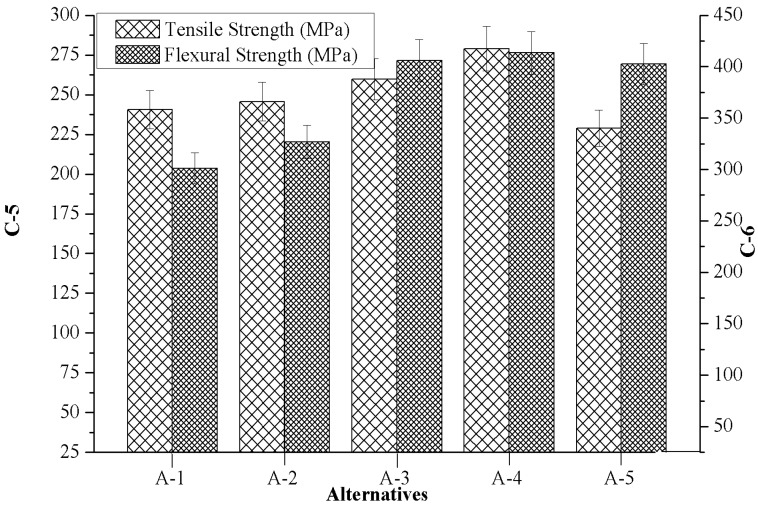
Variation of criterion-5 and criterion-6 with the values of alternatives.

Figure 5 indicates the effects of marble reinforcement on criterion-5 (tensile strength) and criterion-6 (flexural strength) of composites. It was observed that both criterion-5 and criterion-6 were significantly altered with the addition of marble particulates. From Figure 5 it can also be seen that the composite with 4.5 wt.% marble content (A-4) showed the highest tensile as well as flexural strength; however, the lowest tensile and flexural strength were observed for the composite reinforced with 6 wt.% (A-5) and 0 wt.% (A-1) marble content. The lower value of tensile and flexural strength of composites reinforced with marble particulate can be attributed to (a) the increasing brittleness of composites at a higher weight of reinforcement, (b) the presence of casting defects, such as porosity, microcracks, and the clustering of marble particulates, and (c) the poor interface bonding strength between the matrix alloy and the reinforcement.

### 4.4. Effect of Marble Dust Reinforcement on Criterion-7 and Criterion-8

**Figure 6:**
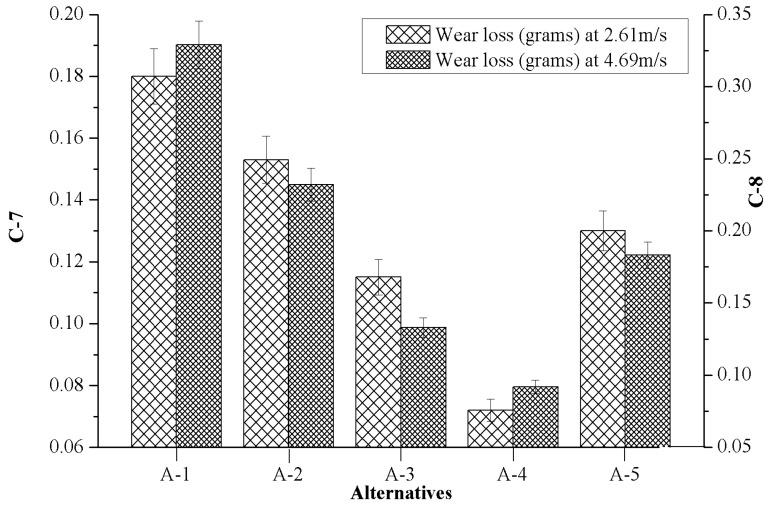
Evolution of criterion-7 together with criterion-8 in respect to the values of alternatives.

Figure 6 presents the effects of marble reinforcement on wear loss with respect to varying sliding velocity 2.61 m/s (C-7) and 4.69 m/s (C-8) while keeping the normal load 15N constant. From Figure 6 it can be appreciated that the unfilled matrix alloy (A-1) showed the highest wear loss whereas the composite with 4.5 wt.% of marble content (A-4) showed the lowest wear loss for both (C-7 and C-8) the test conditions. When unfilled matrix alloys pins were run separately at sliding velocity of 2.61 m/s and 4.69 m/s, the highest wear loss was observed at a sliding velocity of 4.69 m/s. During the experiment it was also seen that material was removed by a ploughing mechanism. When ductile material (i.e., C93200 alloy) slid over the counterface (EN-31 hardened steel disc) at a high load and sliding velocity, its ductility increased due to high frictional heat, and the unfilled composites’ pin surface was ploughed by the hard and sharp asperities of the counterface. Jin et al. [33] performed the sliding wear test on Mg_2_B_2_O_5_ whisker reinforced 6061 Al matrix composite, and reported a similar observation in their investigation. Similarly, when composites reinforced with 4.5 wt.% (A-4) marble content were run at a sliding velocity of 2.61 m/s, less wear loss was observed, but when the same composite was run at a higher sliding velocity (4.69 m/s) a slightly higher wear loss was observed. The decrement in the wear loss may be due to the incorporation of hard particulates and good bonding strength at the interface of matrix alloy with the hard particulates, which may promote a higher hardness features and good wear resistance of composites. 

### 4.5. Effect of Marble Dust Reinforcement on Criterion-9 and Criterion-10

**Figure 7:**
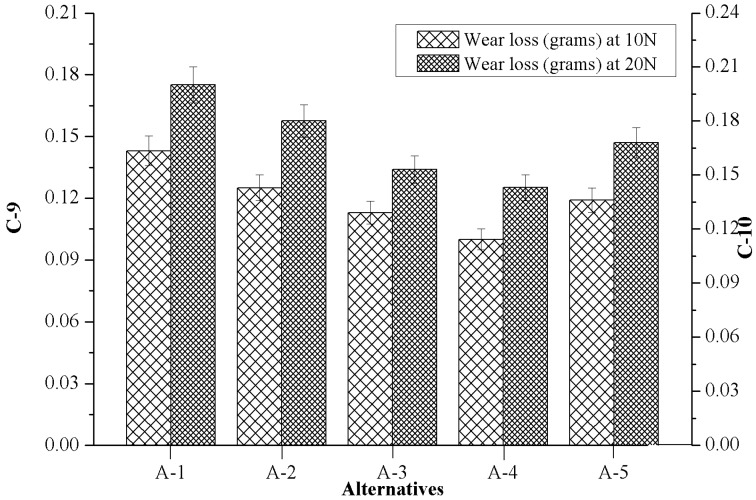
Variation of criterion-9 and criterion-10 with the values of alternatives.

Figure 7 shows the influence of marble dust reinforcement on wear loss in respect to normal load 10N (C-9) and 20N (C-10), while the sliding velocity was kept constant at 3.66 m/s. From Figure 7 it can be noticed that the unreinforced matrix alloy (A-1) showed the highest wear loss, whereas the composite reinforced with 4.5 wt.% of marble particulate (A-4) showed the lowest wear loss for both (C-9 and C-10) test conditions. It was also found that wear loss was higher when it was increased to the normal load for all the alternatives. Similar observations are also reported by Aherwar et al. [34] for Co30Cr4Mo biomedical alloy when nickel was added. Furthermore, the authors observed that with the increase in load on the pins in the actual area of contact could lead to the extension of the nominal area of contact, in which case the frictional force increased. The higher frictional values along the novel surface of contact that were generated were responsible for a large amount of wear loss. Further, it was also found that the composite having a high hardness showed a low wear loss. The reason for the subsidence in wear loss with respect to hardness is that the soft matrix alloy which surrounds the hard reinforced particles was soon worn out due to two body abrasion, which left the hard reinforced particles exposed to the counter body and the applied normal load was transferred to the exposed hard particles which increased the wear resistance of composites.

### 4.6. Effect of Marble Dust Reinforcement on Criterion-11 and Criterion-12

**Figure 8:**
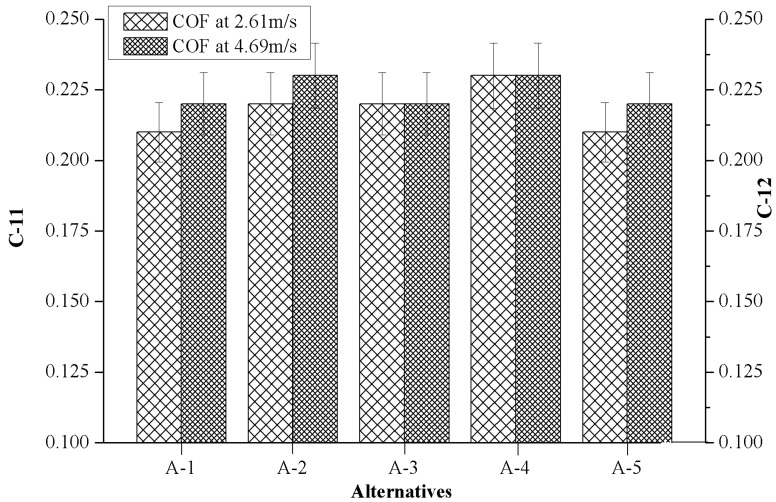
Evolution of criterion-11 and criterion-12 with the values of alternatives.

The effects of marble dust reinforcement on criterion-11 (COF 2.61 m/s, 15 N) and criterion-12 (COF 4.69 m/s, 15 N) of composites are depicted in Figure 8. It can be appreciated that the value of COF (µ) varies in a very narrow range (0.21 ≤ µ ≤ 023) among all the values of alternatives. Further, when the weight percentage of marble reinforcement was increased, a higher friction coefficient was noted. This later circumstance occurred during the addition of hard marble particulates which enhanced the abrasive elements in the present investigated composites. The maximum value (i.e., 0.23) of COF was observed in A-4 for both the test conditions. It was also found that slightly higher COF was observed at higher sliding velocity (4.69 m/s) as compared to COF at lower sliding velocity (2.61 m/s).

### 4.7. Effect of Marble Dust Reinforcement on Criterion-13 and Criterion-14

**Figure 9:**
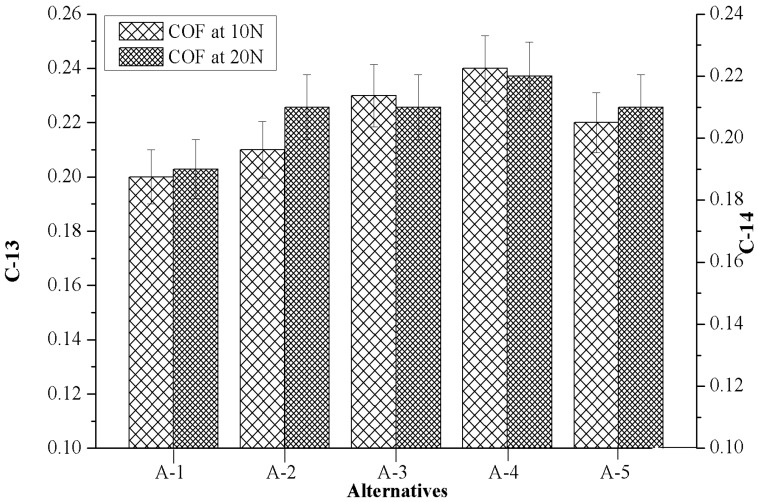
Evolution of criterion-13 together with criterion-14 in respect to the values of alternatives.

Figure 9 depicts the influences of marble dust reinforcement on criterion-13 (COF at 10 N, 3.66 m/s) and criterion-14 (COF at 20 N, 3.66 m/s) of composites. It reveals higher friction values, with a higher (>0.20) magnitude of COF that was observed when the composite ran according to C-13 test conditions, and the maximum COF (0.24) was observed for A-4. It was also seen that the almost constant magnitude of COF was observed when composites ran according to the C-14 test condition and the least COF was observed for A-1.

### 4.8. Identification of the Final Rank with Respect to Various Criteria

Table 3 and Figure 3, Figure 4, Figure 5, Figure 6, Figure 7, Figure 8 and Figure 9 prove the lack of a single alternative to meet the excellence, in terms of performance, motivated by simultaneous combinations of criteria associated with the composite manufactured. In such scenarios, the PSI technique plays a vital role in finding the optimal alternatives. The data obtained in Table 3 were produced using Equation (4), data which permits creating the decision matrix. To improve the compatibility of this decision matrix, the values gathered as alternatives in Table 3 were normalized using Equation (5) in the range of 0–1 and then the results were inserted in Table 4. The normalized matrix, the deviation (Ω*_j_*) from the preference variation value (Ψ*_j_*) together with the overall preference value (Φ*_j_*), and preference selection index value (Π*_j_*) was computed by using Equations (6)–(9) (listed in Table 5) and the alternative with the highest Π*_j_* value (Table 6) were considered as the best alternative. It was examined that the PSI (Π*_j_*) obtained from the A-4 data reached the maximum (0.9607). The following detected values are represented by the A-3 (0.9008), A-5 (0.8467) and A-2 (0.8326), respectively. The material manufactured for bearing, denoted as A-1, presents the least favored condition that has an index of 0.8059. The final optimal sequence of investigated composites alternative raking is ordered as A-4 (Rank-1) > A-3 (Rank-2) > A-5 (Rank-3) > A-2 (Rank-4) > A-1 (Rank-5). 

To validate that our proposed optimization method was suited for automotive component material selection, two different methodologies reported in the literature are used as a basis for comparison and proof that the proposed methodology has a successful implementation. The present methodology i.e., the PSI technique is compared with GRA and the TOPSIS approaches [14,35], as presented in Table 7. The Table 7 shows that the ranking order obtained by the proposed methodology (PSI) of the alternative sets is A-4 > A-3 > A-5 > A-2 > A-1 whereas A-4 > A-3 > A-2 > A-1 > A-5 and A-4 > A-3 > A-2 > A-5 > A-1 ranking order obtained when solved with TOPSIS and GRA methods respectively. This means material alternative A-4, i.e., 4.5 wt.% marble dust reinforcement is the first choice, and A-3 i.e., 3 wt.% marble dust reinforcement, A-5 i.e., 6 wt.% marble dust reinforcement, A-2 i.e., 1.5 wt.% marble dust reinforcement and A-1 i.e., 0 wt.% marble dust reinforcement are located as the second, third, fourth, and fifth choices, respectively. Further, the TOPSIS method suggests material alternative A-4, i.e., 4.5 wt.% marble dust reinforcement is the first choice, and A-3 i.e., 3 wt.% marble dust reinforcement, A-2 i.e., 1.5 wt.% marble dust reinforcement, 0 wt.% marble dust reinforcement and 6 wt.% marble dust reinforcement are located as the second, third, fourth, and fifth selection, respectively. Whereas, the GRA method suggests material alternative A-4, i.e., 4.5 wt.% marble dust reinforcement is the first choice, and A-3 i.e., 3 wt.% marble dust reinforcement, A-2 i.e., 1.5 wt.% marble dust reinforcement, A-5 i.e., 5 wt.% marble dust reinforcement and A-1 i.e., 6 wt.% marble dust reinforcement are located as the second, third, fourth and fifth selection, respectively. All investigated methods prove that the best material alternative A-4, i.e., 4.5 wt.% marble dust reinforcement is the first choice for the given application. Thus, it can be concluded that the ranking of automotive component materials may be predicted with appreciable accuracy by PSI technique.

## 5. Surface Morphology

**Figure 10:**
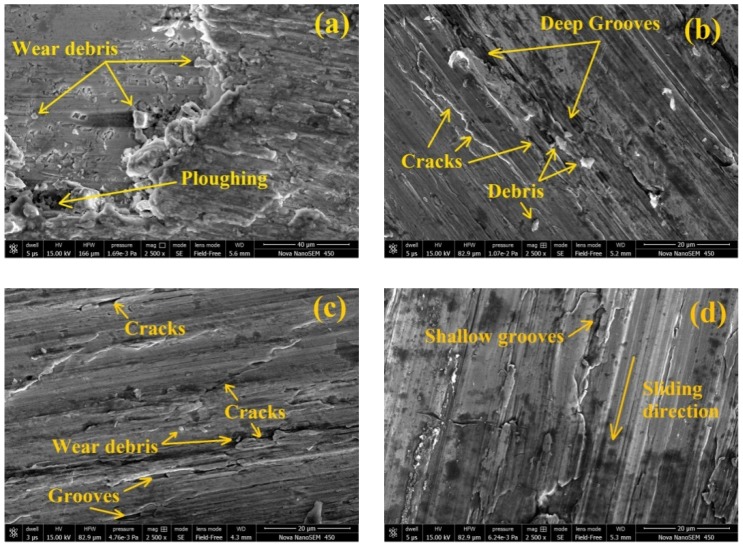

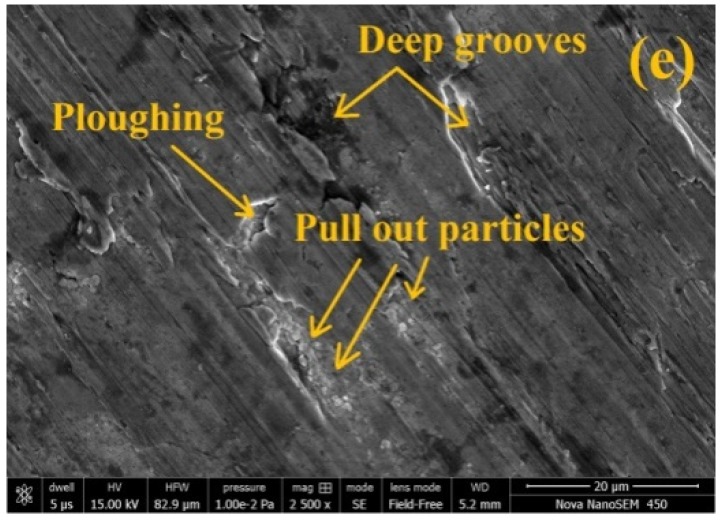
Representative micrographs of selected composite after wear test under 15-N normal load, 4.69 m/s sliding velocity and 1500 m sliding distance of (**a**) A-1, 0 wt.% MD; (**b**) A-2, 1.5 wt.% MD; (**c**) A-3, 3 wt.% MD; (**d**) A-4, 4.5 wt.% MD; (**e**) A-5, 6 wt.% MD reinforced composites.

SEM micrographs of C93200 alloy (A-1) and marble dust reinforced composites (A-2, A-3, A-4 and A-5) after the wear tests at 4.69 m/s sliding velocity under 15N normal load and 1500 m sliding distance are shown in Figure 10. The sliding direction traces, wear debris, surface micro cracks and grooves, material removal, and ploughing strips were observed on the samples’ surfaces after wear tests.

The SEM image of the control’s condition (unreinforced marble dust; A-1) (see Figure 10a) indicate that the presence of the reinforcement material had become negligible. The surface micrograph clearly confirmed that ploughing (abrasion) was the main cause of material removal. The base matrix (C93200) alloy proved ductile material properties, whereby their ductility may have increased with the sliding contact over the counter body when a higher load was imposed; and the sliding velocity generated frictional heat that promoted a softer matrix material that ploughed into the hard asperities of the counterface. Figure 10b shows the surface micrograph of the A-2 composite where some cracks and continuous grooves were observed. The main causes of producing the continuous grooves were estimated to be (1) due to hard asperities penetration (two body abrasions) and (2) the abrasions were responsible for the fractured hard reinforced particles (three body abrasions). Figure 10c shows the micrograph of A-3 composites where macro cracks, together with a delamination wear mechanism and dense distribution of marble particulates were observed. Figure 10d shows the surface micrograph where shallow grooves along the sliding direction were observed for A-4 composites. Among all the fabricated composites Figure 10d shows the lowest wear loss for A-4 composites, and the decrease in wear loss may be because of proper filler wt.% content bonding between hard reinforcement particles and base matrix alloy which resulted in the high hardness of composites. Under similar operating conditions, the removal of the matrix material was observed for A-5 composites as shown in Figure 10e. At 4.69 m/s sliding velocity and 15 N load, a pin slid over the counterface and a substantial amount of frictional heat was generated. This frictional heat allowed for a softening of the material matrix and lead to a lower interfacial bonding strength that occurred at the interface between marble dust and copper alloy, which may have resulted in specific hard reinforced particles (i.e., marble dust) being pulled out, which resulted from the softened matrix alloy.

## 6. Conclusions

The present paper studied a detailed experimental investigation in order to develop a novel composite material for bearing application. In the primary stage, the novel manufactured composite material formed from copper-based (C93200) metal alloy mixed with different weight percentages of marble dust was characterized for its physical, mechanical, and tribological performances. The tests proved that the inclusion of marble dust allowed to improve their physical, mechanical, and tribological features of the novel bearing materials manufactured. The addition of 4.5 wt.% marble dust in the matrix material (i.e., A-4) revealed an effective improvement in terms of wear resistance when it was submitted to different loading conditions. Here, the PSI method proposed permitted us to well define the final ranking for the material developed which is categorized as A-4 > A-3 > A-5 > A-2 > A-1. Because the A-4 demonstrated the highest ranking (i.e., 0.9607) on the preference selection index technique, it can be considered amongst the best candidates and a promising composite for bearing applications.

## Figures and Tables

**Table 1:** Detailed composition of composites.

Sample Designateon	Composition
A-1	C93200 copper alloy + 0 wt.% Marble dust
A-2	C93200 copper alloy + 1.5 wt.% Marble dust
A-3	C93200 copper alloy + 3 wt.% Marble dust
A-4	C93200 copper alloy + 4.5 wt.% Marble dust
A-5	C93200 copper alloy + 6 wt.% Marble dust

C93200 copper alloy composition: Cu ball, 7 wt.% Sn, 6 wt.% Pb, 2 wt.% Zn, 1 wt.% Ni, 0.5 wt.% Fe.

**Table 2:** Details from the selected criteria implemented.

Criterions		Test Conditions	Performance Implications
C-1:	Density (gm/cc)	Archimedes method	Min
C-2:	Void content	-	Min
C-3:	Hardness (Hv)	ASTM E-92	Max
C-4:	Compressive strength (MPa)	ASTM E 209-00	Max
C-5:	Tensile strength (MPa)	ASTM E 209-00	Max
C-6:	Flexural strength (MPa)	ASTM E 209-00	Max
C-7:	Wear loss (grams)	15 N ^1^; 2.61 m/s ^2^	Min
C-8:	Wear loss (grams)	15 N ^1^; 4.69 m/s ^2^	Min
C-9:	Wear loss (grams)	10 N ^1^; 3.66 m/s ^2^	Min
C-10:	Wear loss (grams)	20 N ^1^; 3.66 m/s ^2^	Min
C-11:	COF (µ)	15 N ^1^; 2.61 m/s ^2^	Min
C-12:	COF (µ)	15 N ^1^; 4.69 m/s ^2^	Min
C-13:	COF (µ)	10 N ^1^; 3.66 m/s ^2^	Min
C-14:	COF (µ)	10 N ^1^; 3.66 m/s ^2^	Min

^1^ Normal load; ^2^ Sliding velocity.

**Table 3:** Experimental results of the criteria.

Composite	C-1	C-2	C-3	C-4	C-5	C-6	C-7	C-8	C-9	C-10	C-11	C-12	C-13	C-14
A-1	8.84	0.785	115.49	36.68	240.71	301.02	0.180	0.329	0.143	0.200	0.21	0.22	0.20	0.19
A-2	8.55	0.696	116.73	40.47	245.67	326.60	0.153	0.232	0.125	0.180	0.22	0.23	0.21	0.21
A-3	8.26	0.601	121.51	40.60	259.73	406.03	0.115	0.133	0.113	0.153	0.22	0.22	0.23	0.21
A-4	8.00	0.497	128.97	39.32	278.99	413.34	0.072	0.092	0.100	0.143	0.23	0.23	0.24	0.22
A-5	7.63	1.548	120.94	38.32	228.97	402.44	0.130	0.183	0.119	0.168	0.21	0.22	0.22	0.21

**Table 4:** Normalized decision matrix.

Composite	C-1	C-2	C-3	C-4	C-5	C-6	C-7	C-8	C-9	C-10	C-11	C-12	C-13	C-14
A-1	0.863	0.633	0.895	0.903	0.863	0.728	0.402	0.280	0.699	0.715	1.000	1.000	1.000	1.000
A-2	0.892	0.714	0.905	0.997	0.881	0.790	0.473	0.397	0.800	0.794	0.955	0.957	0.952	0.905
A-3	0.924	0.827	0.942	1.000	0.931	0.982	0.629	0.692	0.885	0.935	0.955	1.000	0.870	0.905
A-4	0.954	1.000	1.000	0.968	1.000	1.000	1.000	1.000	1.000	1.000	0.913	0.957	0.833	0.864
A-5	1.000	0.321	0.938	0.944	0.821	0.974	0.556	0.503	0.840	0.851	1.000	1.000	0.909	0.905

**Table 5:** The preference variation (Ψ*_j_*), deviation related to the preference variation (Ω*_j_*), and the overall preference value (Φ*_j_*).

Composite	C-1	C-2	C-3	C-4	C-5	C-6	C-7	C-8	C-9	C-10	C-11	C-12	C-13	C-14
Ψ*_j_*	0.863	0.633	0.895	0.903	0.863	0.728	0.402	0.280	0.699	0.715	1.000	1.000	1.000	1.000
Ω*_j_*	0.892	0.714	0.905	0.997	0.881	0.790	0.473	0.397	0.800	0.794	0.955	0.957	0.952	0.905
Φ*_j_*	0.924	0.827	0.942	1.000	0.931	0.982	0.629	0.692	0.885	0.935	0.955	1.000	0.870	0.905

**Table 6:** The preference selection index (Π*_j_*) and its alternatives rankings.

Alternative	Preference Selection Index (Π*_j_*)	Ranking
A-1	0.8059	5
A-2	0.8326	4
A-3	0.9008	2
A-4	0.9607	1
A-5	0.8467	3

**Table 7:** Comparison of proposed methodology with TOPSIS and GRA.

Alternatives	Ranking		
	Proposed	TOPSIS	GRA
A-1	5	4	5
A-2	4	3	3
A-3	2	2	2
A-4	1	1	1
A-5	3	5	4
